# Dynamics of Small RNA Profiles of Virus and Host Origin in Wheat Cultivars Synergistically Infected by *Wheat Streak Mosaic Virus* and *Triticum Mosaic Virus*: Virus Infection Caused a Drastic Shift in the Endogenous Small RNA Profile

**DOI:** 10.1371/journal.pone.0111577

**Published:** 2014-11-03

**Authors:** Satyanarayana Tatineni, Jean-Jack M. Riethoven, Robert A. Graybosch, Roy French, Amitava Mitra

**Affiliations:** 1 United States Department of Agriculture-Agricultural Research Service (USDA-ARS) and Department of Plant Pathology, University of Nebraska-Lincoln, Lincoln, Nebraska, United States of America; 2 Center for Biotechnology, University of Nebraska-Lincoln, Lincoln, Nebraska, United States of America; 3 USDA-ARS and Department of Agronomy and Horticulture, University of Nebraska-Lincoln, Lincoln, Nebraska, United States of America; 4 USDA-ARS, University of Nebraska-Lincoln, Lincoln, Nebraska, United States of America; 5 Department of Plant Pathology, University of Nebraska-Lincoln, Lincoln, Nebraska, United States of America; University of Basel, Switzerland

## Abstract

Co-infection of wheat (*Triticum aestivum* L.) by *Wheat streak mosaic virus* (WSMV, a *Tritimovirus*) and *Triticum mosaic virus* (TriMV, a *Poacevirus*) of the family *Potyviridae* causes synergistic interaction. In this study, the effects of the synergistic interaction between WSMV and TriMV on endogenous and virus-derived small interfering RNAs (vsiRNAs) were examined in susceptible (‘Arapahoe’) and temperature-sensitive resistant (‘Mace’) wheat cultivars at 18°C and 27°C. Single and double infections in wheat caused a shift in the profile of endogenous small RNAs from 24 nt being the most predominant in healthy plants to 21 nt in infected wheat. Massive amounts of 21 and 22 nt vsiRNAs accumulated in singly and doubly infected Arapahoe at both temperatures and in Mace at 27°C but not 18°C. The plus- and minus-sense vsiRNAs were distributed throughout the genomic RNAs in Arapahoe at both temperature regimens and in Mace at 27°C, although some regions served as hot-spots, spawning an excessive number of vsiRNAs. The vsiRNA peaks were conserved among cultivars, suggesting that the Dicer-like enzymes in susceptible and resistant cultivars similarly accessed the genomic RNAs of WSMV or TriMV. Accumulation of large amounts of vsiRNAs in doubly infected plants suggests that the silencing suppressor proteins encoded by TriMV and WSMV do not prevent the formation of vsiRNAs; thus, the synergistic effect observed is independent from RNA-silencing mediated vsiRNA biogenesis. The high-resolution map of endogenous and vsiRNAs from WSMV- and/or TriMV-infected wheat cultivars may form a foundation for understanding the virus-host interactions, the effect of synergistic interactions on host defense, and virus resistance mechanisms in wheat.

## Introduction

Co-infection of plants with two or more viruses is common in nature because plants are susceptible to multiple viruses. Additionally, many hemipteran vectors transmit more than one virus, thus causing mixed infections by related or unrelated viruses [1], leading to antagonistic or synergistic interactions [2]. Antagonistic interactions result from infection of plants by a virus, followed by different strains of the same virus at different times, where only one virus strain is the beneficiary, and its preexistence prevents superinfection by a second virus [3], [4]. In contrast, synergistic interactions among unrelated viruses in mixed infections display a facilitative effect on one or both of the viral partners, with more severe symptoms than those induced by individual viruses [5]–[11]. Synergistic interactions among some other virus combinations have been reported as host-dependent [8], [11]–[13], suggesting that in addition to interactions among viral partners, successful interaction between viral and host proteins might play an important role in synergistic interactions. However, several aspects of synergistic interactions are not clearly understood. For example, how does co-infection by two unrelated viruses cause more severe symptoms than infection by individual viruses? What are the effects of virus-encoded RNA silencing suppressor proteins on host defense mechanisms in co-infected plants compared with plants infected by individual viruses?

Plant viruses are considered to be strong inducers as well as targets of host RNA silencing. RNA silencing is one of the most important antiviral defense mechanisms used by plants against viral infections [14], [15]. In addition to antiviral defense, RNA silencing regulates several plant developmental processes, transposon control, DNA methylation, and chromatin modification [16], [17]. Viral double-stranded (ds) RNAs, generated by viral RNA-dependent RNA polymerases as replication-intermediate forms or as highly structured portions of single-stranded viral genomic and sub-genomic RNAs, trigger the antiviral RNA silencing defense mechanism in plants [18]–[22]. These dsRNAs are recognized by Dicer-like (DCL) ribonucleases and are processed into 21 to 24 nucleotide (nt) short dsRNAs known as virus-derived small interfering RNAs (vsiRNAs) [23]–[27]. Two classes of vsiRNAs have been identified in virus-infected plants, namely, primary vsiRNAs derived from the initial cleavage of virus derived dsRNA-like structures by DCLs, and secondary vsiRNAs formed by amplification of primary vsiRNAs by host RNA-dependent RNA polymerases (RDRs) [24], [28]. These vsiRNAs are recruited by Argonaute (AGO) proteins to form a multiprotein effector complex, an RNA-induced silencing complex (RISC), and guide the cleavage of homologous RNAs in a sequence-specific manner, or induce translational repression or chromatin modification [29]–[32].

Both monocotyledonous and dicotyledonous plants contain DCL ribonucleases, AGOs and RDRs, the core components of host RNA silencing [30]. In *Arabidopsis thaliana* (L.) Heynh, 4 DCLs, 10 AGOs, and 6 RDRs have been identified [33]. Among monocots, the RNA silencing phenomenon and its components have extensively been studied only in rice (*Oryza sativa* L.). So far, in rice, 8 DCLs, 19 AGOs and 5 RDRs have been identified [34]. The presence of different numbers of RNA silencing core components in rice and *A. thaliana* suggests that different RNA silencing pathways might be utilized by these two angiosperms. The mechanisms of RNA silencing were extensively studied in *A. thaliana* using mutant plants that defined the roles of DCLs, AGOs and RDRs in RNA silencing [35]. In addition to the processing of endogenous precursor RNAs to sRNAs, all four DCLs found in *A. thaliana* have been implicated in the formation of vsiRNAs [36], [37]. However, their action is hierarchical, with DCL4 and DCL2 being the main contributors [23]. Loading vsiRNAs on to the AGO complex in plants is preferentially, but not exclusively, dictated by the 5′-terminal nucleotide of vsiRNAs [38].

Wheat (*Triticum aestivum* L.) is the world’s most widely grown crop, covering 200 million hectares with an annual production of 681 million tons in 2011, slightly behind maize (*Zea mays* L.) and rice, providing the primary source of carbohydrates, proteins, vitamins, and minerals for the world’s population [39]. The 17-gigabase-pair hexaploid wheat genome is complex, about three times larger than the human genome [40], [41]. Among viral pathogens infecting wheat in the Great Plains region of the United States, the main U.S. wheat-growing region, *Wheat streak mosaic virus* (WSMV) is the most economically important virus causing significant yield losses [42], [43]. Recently, *Triticum mosaic virus* (TriMV), a new virus, was reported from several Great Plains states [44]–[46]. WSMV and TriMV are the type members of the *Tritimovirus* and *Poacevirus* genera, respectively, in the family *Potyviridae* [47], [48]. Both of these viruses are vectored by *Aceria tosichella* Keifer, the wheat curl mite [49]–[51], resulting in mixed infections in growers’ fields [44], [52]. Wheat cultivar (cv.) Mace is resistant to WSMV at temperatures below 19°C [53]. Mace’s resistance to WSMV is conditioned by the *Wsm*-1 gene, which is also found to provide similar levels of resistance to TriMV [8], [52]. *Wsm-1* was introduced to wheat from the related perennial grass *Thinopyrum intermedium* (Host) Barkworth & D.R. Dewey [54].

WSMV and TriMV possess limited sequence homology but interact synergistically in co-infected susceptible wheat cvs. Arapahoe and Tomahawk at 19°C and 20 to 26°C, resulting in increased accumulation of both viruses and enhanced disease severity [8], [52]. However, in Mace, double infections induced moderate disease synergism at 20 to 26°C but not at 19°C. The P1 proteins of WSMV and TriMV have been reported to be involved in the suppression of local and systemic RNA silencing in *N. benthamiana* 16c plants [55], [56]. However, the response of wheat defense mechanisms against single and double infections in susceptible and resistant wheat cultivars with WSMV and TriMV has not been examined, and it is not known how viral infection(s) trigger cascades of host defense mechanisms that cause production of vsiRNAs, an important component of host RNA silencing.

In this study, the dynamics of endogenous sRNAs and vsiRNAs were examined in WSMV and/or TriMV-infected susceptible and temperature-sensitive resistant wheat cultivars as a first step toward understanding virus-host interactions in synergistic infections.

## Materials and Methods

### Virus source

*In* *vitro* transcripts from an infectious cDNA clone of WSMV isolate Sidney 81 were inoculated onto wheat (cv. Tomahawk) seedlings at the single leaf stage [57]. A TriMV Nebraska isolate was activated from dried wheat leaves that were stored at 4°C in a 125-ml Erlenmeyer flask over anhydrous calcium sulfate [48] by inoculating wheat seedlings at the single-leaf stage. WSMV, TriMV and WSMV plus TriMV inocula were prepared from 12 dpi wheat cv. Arapahoe infected with WSMV or TriMV at 1∶20 dilution in 20 mM sodium phosphate buffer, pH 7.0, and mechanically inoculated to wheat cvs. Arapahoe and Mace at the single leaf stage, two pots per inoculum with 12 to 15 seedlings per pot. Inoculated wheat plants were incubated in growth chambers (Percival, Perry, IA) at 18°C and 27°C with a 16 h photoperiod. Fully expanded upper leaves of infected wheat at 16 dpi were harvested for total RNA extraction.

### Isolation of total RNA

One gram of fully expanded upper leaves of wheat infected with WSMV, TriMV, WSMV plus TriMV, or healthy plants was ground in liquid nitrogen into a fine powder, followed by the addition of 5 ml of TriPure isolation reagent (Roche, Indianapolis, IN). The tissue was ground thoroughly and the extract was transferred to an Oak ridge tube. The mortar was washed with an additional 2 ml of TriPure isolation reagent. The extract was vortexed for 30 s, followed by the addition of 1.4 ml of chloroform and vortexed for 30 s. This mixture was incubated at room temperature for 10 min, followed by clarification at 12,000×*g* for 15 min at 4°C. Total RNA was precipitated from the 3 ml aqueous phase by adding an equal volume of isopropanol and incubated at room temperature for 10 min. The RNA was pelleted at 12,000×*g* for 10 min at 4°C, and the pellet was washed with 5 ml of 70% ethanol. The air-dried RNA pellet was suspended in 500 µl of sterile water and stored frozen at –70°C. The amount of total RNA was quantified using a NanoPhotometer (Implene Inc., Westlake Village, CA).

### Northern blot hybridization

Two µg of total RNA was separated on 15% polyacrylamide-urea gels (Bio-Rad, Hercules, CA), followed by electro-transfer to Nylon membranes (Roche). Nylon membranes were probed with DIG-labeled minus-strand RNA riboprobes specific to nt 6303 to 9384 of WSMV or nt 6401 to 10,245 of TriMV. These probes were further hydrolyzed into ∼50 nt long RNA pieces by treatment with sodium carbonate as described in Dalmay et al. [58]. Prehybridization and hybridization were carried out essentially as described in Tatineni et al. [55].

### Isolation of small RNAs

Thirty µg of total RNA was denatured in the presence of 47.5% formamide, 0.1X TBE buffer plus 0.015% of each bromophenol blue and xylene cyanol FF at 95°C for 4 min, followed by incubation on ice for 5 min. Denatured total RNA was separated on a 15% polyacrylamide-urea-TBE gel (Bio-Rad) at 120 V until the bromophenol blue reached the bottom of the gel. Gel slices from just below the center of the xylene cyanol band and extending to three-quarters of the way toward the bromophenol blue band were collected into a 30 ml corex tube containing 5 ml of 0.3 M sodium chloride. The gel slices were crushed into small pieces with a 10 ml syringe piston and incubated in a shaker at 4°C overnight. The supernatant was collected after centrifugation at 10,000 g for 15 min at 4°C. Two ml of 0.3 M sodium chloride was added to acrylamide pieces and incubated in a shaker for 1 h at room temperature. The small RNAs were precipitated from the pooled supernatants by addition of 3 volumes of ice-cold ethanol in the presence of 0.3 M sodium acetate, pH 5.2 and 2 µl of glycogen (Roche), and incubated at −70°C for 1.5 h. The small RNAs were suspended in 350 µl of 0.3 M sodium chloride and loaded onto a spin-x-cellulose acetate filter (Sigma-Aldrich, St. Louis, MO). The spin column was centrifuged at 10,000 g for 2 min at room temperature. The small RNAs were precipitated from the elutant by adding 3 volumes of ethanol in the presence of 0.3 M sodium acetate and 1 µl glycogen for 30 min at −70°C, followed by centrifugation at 12,000 g for 15 min at 4°C. The RNA pellet was washed with 70% ethanol, and the air dried small RNA pellet was suspended in 10 µl sterile water and stored at −70°C.

### Deep sequencing

Small RNA libraries were prepared from gel-eluted small RNA fractions of total RNA using Illumina TruSeq small RNA Sample Prep Kit (Illumina Inc, San Diego, CA), essentially as described in the manufacturer’s instructions at the University of Nebraska-Lincoln Genomics Core Research Facility. Illumina TruSeq SR Cluster Generation Kit v5 was used to generate the cluster at one sample per lane. Amplified library was sequenced on Illumina Genome Analyzer IIx using a 36-cycle sequencing kit to read 36 nts of sequence from a single end of each insert by standard v8 protocol. The raw sequence reads for all 16 samples were deposited in the GEO Omnibus under accession GSE54026.

### Bioinformatics analysis of small RNAs

Residual Illumina TruSeq adapters were removed from the small RNA samples via the *cut-adapt* tool (version 0.9.5, [59]) using the following parameters: a maximum error rate of 15% (−e 15), base trimming when the Phred-like quality score was lower than 45 (−q 45), and iterative removal of adapters up to three times (−n 3). Thus, the cleaned 16 samples contained a grand total of 14 billion bases spanning approximately 570 million reads (Table S1). The number of reads found in healthy and infected wheat cultivars was normalized against the reads of healthy Arapahoe at 18°C (Table S2). The initial size distribution of the small RNAs were determined for each sample via a custom in-house Perl script.

Both healthy and virus-infected samples were mapped via bowtie1 (version 0.12.8, [60]) against the genomes of both WSMV (Ref Seq NC_001886.1) and TriMV (Ref Seq NC_012799.1) to determine the amount of virus-derived small RNAs. The alignment parameters for bowtie1 were as follows: a seed length of 25 (−l 25), one mismatch per seed (−n 1), up to 5 alignments per read (−k 5), suppress any read that maps more than 10 times (−m 10), and valid best alignments (–tryhard –best).

The small RNAs were mapped against WSMV and TriMV genome sequences and small RNAs that did not align to these viral sequences were considered to be of host origin. Similarly, cross-contamination of healthy samples with virus was detected and removed. A custom in-house Perl script was developed to target the small RNAs of interest and to generate information on the final length distribution and the nucleotide frequency at the 5′ end of the small RNAs, as well as coverage information on the virus genomes. As extra filters for the final output, in the case of the healthy samples, any read with a non-called base (N) was removed; for both the healthy and virus-infected samples, small RNAs were size-selected between 18 to 26 nucleotides (inclusive) and reads containing alignment mismatches, or that do not map uniquely, were removed. For the samples with the double virus infection, any RNAs that cross match between the two viruses were removed as well.

MATLAB (2012b, The Mathworks, Inc.) was used to generate coverage graphs of the small RNAs on the virus genomes.

Chi-square analysis was used to determine whether the treatments induced statistically significant changes in the distribution or composition of small RNAs. For each cultivar and temperature treatment, the observed distribution (as percent) of sRNA from 20 to 25 nt in healthy tissue was used as the “expected” class in chi-square calculations.

## Results

### Co-infection of wheat cultivars with WSMV and TriMV caused an increase in the accumulation of vsiRNAs

**Figure 1:**
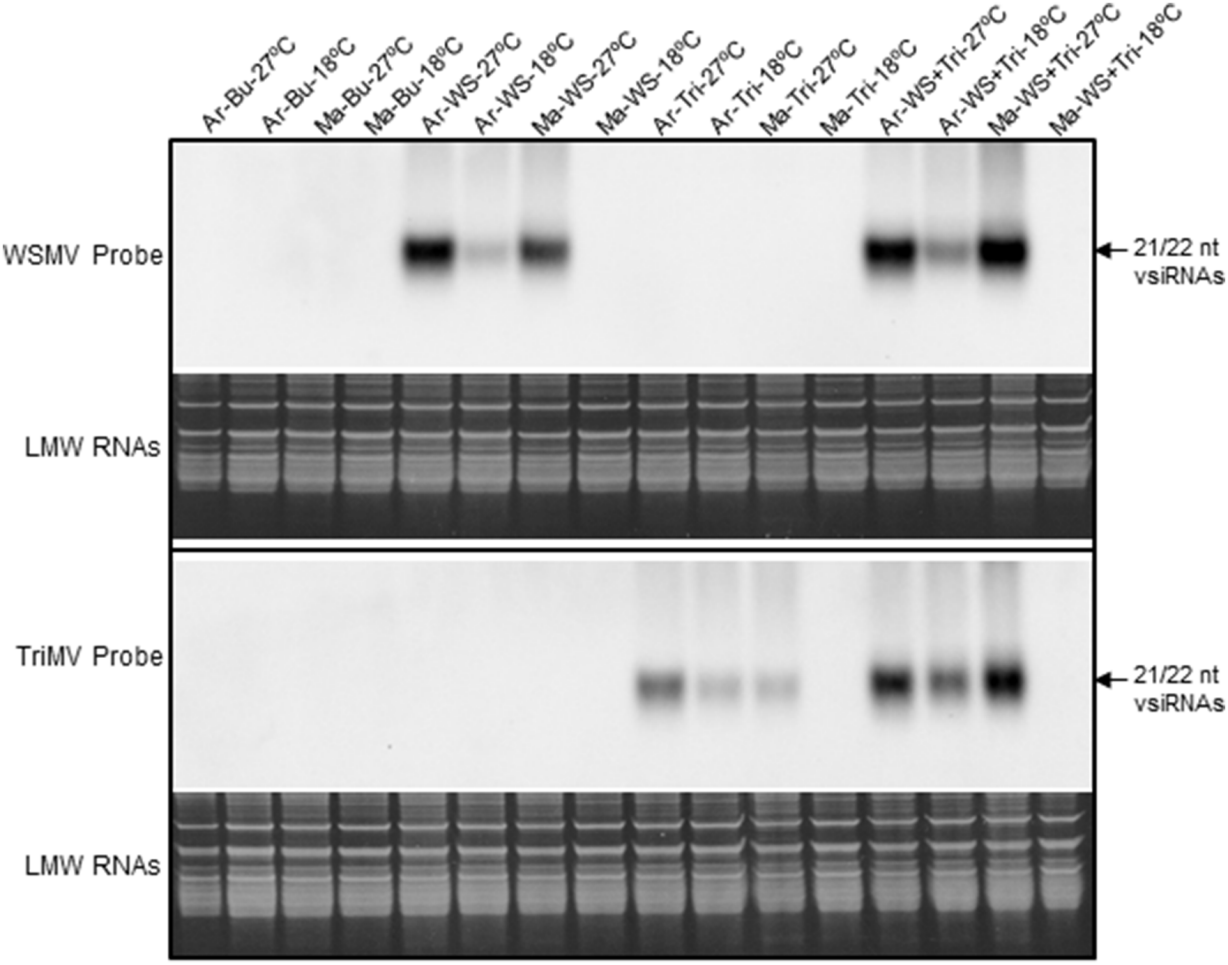
Accumulation of virus-derived small interfering RNAs (vsiRNAs) of plus polarity in WSMV-, TriMV- or WSMV plus TriMV-infected wheat cultivars Arapahoe and Mace at 18°C and 27°C. WSMV and TriMV minus-strand RNA-specific DIG-labeled riboprobes complementary to nucleotides 9384 to 6303 and 10,266 to 6401, respectively, were used for Northern blot hybridization. Riboprobes hydrolyzed to ∼50 nt were used for Northern hybridization of small RNAs. Ethidium bromide stained TBE-urea polyacrylamide gels showing low molecular weight (LMW) RNAs as sample loading control are presented under the Northern blots. Ar: Wheat cv. Arapahoe; Ma: Wheat cv. Mace; WS: WSMV; Tri: TriMV; Bu: buffer.

The effect of the synergistic interaction between WSMV and TriMV on the accumulation of vsiRNAs in wheat cultivars Arapahoe and Mace at 18°C and 27°C was examined by Northern blot hybridization of total RNA using strand-specific digoxigenin (DIG)-labeled riboprobes. The riboprobes hybridized neither with total RNA from healthy wheat cultivars nor another co-infecting virus, suggesting that these riboprobes are highly specific to their respective viruses (Fig. 1). In singly and doubly infected wheat cv. Arapahoe, increased levels of WSMV- and TriMV-specific vsiRNAs accumulated in plants incubated at 27°C compared to 18°C (Fig. 1). In Mace, vsiRNAs were readily detected in singly and doubly infected plants at 27°C but not 18°C, further confirming Mace’s resistance to WSMV and TriMV at 18°C. In wheat cv. Arapahoe at 18°C and 27°C, and cv. Mace at 27°C, increased levels of vsiRNAs accumulated in doubly infected plants relative to levels observed in singly infected plants (Fig. 1), suggesting that synergistic interaction between WSMV and TriMV is not due to the presence of two RNA silencing suppressor proteins.

### High-throughput sequencing of small RNAs from WSMV- and/or TriMV-infected wheat cultivars

To obtain insight into the mechanisms of synergistic interactions among unrelated viruses and the effects of these interactions on host defense mechanisms, the profile of endogenous small RNAs and vsiRNAs was examined from singly (WSMV or TriMV) and doubly (WSMV plus TriMV) infected wheat cultivars Arapahoe (susceptible) and Mace (temperature sensitive-resistant) at 18°C and 27°C.

Composition of small RNAs obtained through Illumina Solexa high-throughput sequencing was examined by computational alignment of sequences identical or complementary to the genomes of WSMV and TriMV. Similarly, small RNAs not corresponding to the viral genomes from doubly infected wheat cv. Arapahoe at 27°C were also BLAST searched for sequences identical to the wheat genome and most of the sRNAs were aligned with the wheat genome. Since there are few gaps in the wheat genome sequence [40], [41], we considered sRNA sequences that do not align with the genomes of WSMV and TriMV as wheat genomic sequences. Moreover, we did not find significant matches to other pathogen sequences present in the database, suggesting that sequences not aligning with WSMV or TriMV most likely represent the wheat genome. Small RNAs between 18 and 26 nt in size were selected for further analyses.

A total of 29.8 to 37.6 million reads were obtained from WSMV- and/or TriMV-infected Arapahoe and Mace at 18°C and 27°C, and 34.2 to 39.2 million reads were obtained from healthy samples (Table 1). In infected samples, 6.8 to 14.3 million size-selected (18 to 26 nt) reads were specific to the wheat genome, in contrast to 10.4 to 13.7 million size-selected reads from healthy samples (Table 1). In healthy samples, only 25 to 1321 virus-specific size selected reads were found, and the authenticity of these reads will be known only after the availability of the complete wheat genome sequence without gaps. In Arapahoe at 18°C and 27°C and Mace at 27°C, 6.5 to 17.1 million reads were aligned with the WSMV or TriMV genome, and 94 to 98% (6.3 to 16.5 million) of these reads were in 18 to 26 nt size class (Table 1). Among size selected reads, 20 to 25 nt sRNAs accumulated at significantly higher levels compared with 18, 19 and 26 nt sRNAs in healthy and virus-infected samples. Hence, further analyses of small RNAs from healthy and virus-infected samples were performed for 20 to 25 nt RNAs.

**Table 1:** Analysis of total small RNA reads from healthy, *Wheat streak mosaic virus* (WSMV) and/or *Triticum mosaic virus* (TriMV) infected wheat cvs. Arapahoe and Mace at 18°C and 27°C.

Sequence reads	Arapahoe 27°C							Arapahoe 18°C						
	Single infection				Double infection			Healthy		Single infection		Double infection		
	Healthy	WSMV	TriMV		WSMV	TriMV				WSMV	TriMV	WSMV	TriMV	
Total # of reads	34.2 M	37.6 M	30.6 M		29.8 M	29.8 M		39.2 M		36.5 M	36.3 M	33.8 M	33.8 M	
Healthy Reads	34.2 M	25.4 M	21.0 M		14.3 M	14.3 M		39.2 M		25.8 M	25.1 M	13.8 M	13.8 M	
Size selected healthy reads (18–26 nt)	13.1 M	14.3 M	8.9 M		7.6 M	7.6 M		13.7 M		11.9 M	11.4 M	7.8 M	7.8 M	
Virus-specific reads	93.8/93.0 K	12.2 M	9.6 M		8.7 M	6.8 M		72.9/73.9 K		10.7 M	11.2 M	11.4 M	8.6 M	
Size selected virus reads (18–26 nt)	55(W)/49(T)	11.9 M	9.4 M		8.4 M	6.6 M		40(W)/29(T)		10.3 M	11.0 M	10.9 M	8.4 M	
Virus-specific exact matches	-	11.5 M	8.9 M		7.7 M	5.9 M		-		10.0 M	10.5 M	10.3 M	7.8 M	
	**Mace: 27°C**							**Mace: 18°C**						
Total # of reads	37.6 M	37.4 M		35.3 M	35.1 M		35.1 M	38.0 M	36.3 M		36.4 M	32.4 M		32.4 M
Healthy Reads	37.6 M	20.3 M		28.8 M	11.1 M		11.1 M	38.0	36.2 M		36.3 M	32.1 M		32.1 M
Size selected healthy reads (18–26 nt)	10.4 M	10.2 M		8.6 M	6.8 M		6.8 M	10.9 M	11.6 M		11.6 M	11.8 M		11.8 M
Virus-specific reads	61.7 K/64.2 K	17.1 M		6.5 M	14.6 M		9.4 M	63.7/65.4 K	116.6 K		106.6 K	178.9 K		118.0 K
Size selected virus reads (18–26 nt)	25(W)/1321(T)	16.5 M		6.3 M	14.0 M		9.2 M	46(W)/432(T)	22.4 K		48.6 K	124.3 K		70.5 K
Virus-specific exact matches	-	15.9 M		5.9 M	13.4 M		8.6 M	-	21.0 K		46.3 K	118.0 K		65.8 K

W: WSMV; T: TriMV; M: millions; K: thousands.

### Virus infection caused a shift in the profile of endogenous sRNAs accumulation

**Figure 2:**
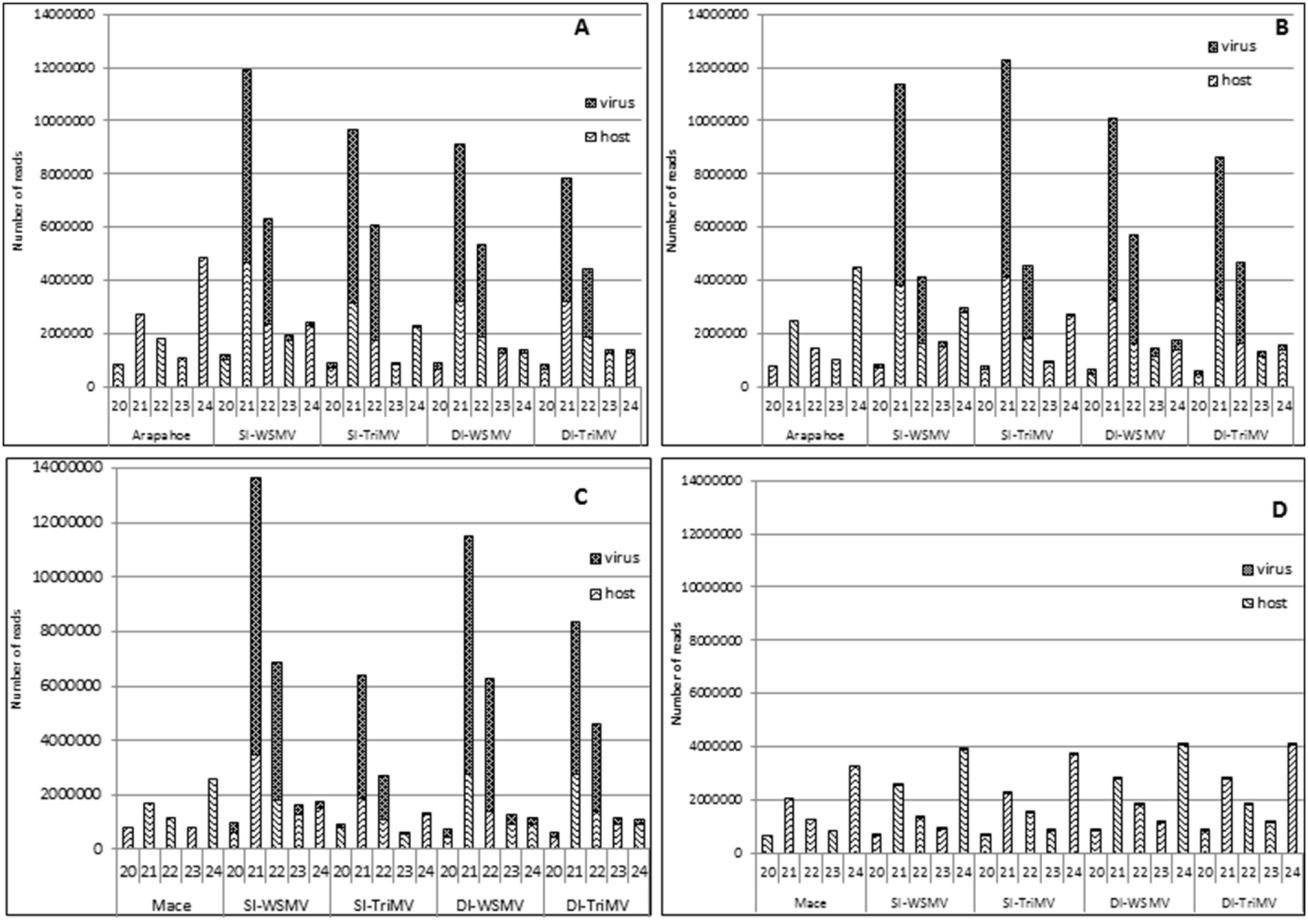
Size distribution of 20 to 24 nt virus- and host-specific small RNAs from WSMV-, TriMV-, or WSMV plus TriMV-infected wheat cvs. Arapahoe and Mace at 18°C and 27°C. A dramatic shift in accumulation of 24 nt endogenous small RNAs (sRNAs) as predominant in healthy samples to 21 nt in singly and doubly infected wheat cvs. Arapahoe at 18°C and 27°C and Mace at 27°C is evident. No shift in predominant sRNA accumulation was observed in wheat cv. Mace at 18°C. SI: single infection; DI: Double infection; A: Arapahoe at 27°C; B: Arapahoe at 18°C; C: Mace at 27°C; D: Mace at 18°C.

In healthy wheat cultivars Arapahoe and Mace at 18°C and 27°C, 24 nt endogenous sRNAs were the most predominant (33 to 41%), followed by 21 nt (22 to 24%), 22 nt (13 to 15%), 23 nt (9 to 10%), and 20 nt (7 to 10%) (Table 2). Among endogenous sRNAs, the predominant 24 nt sRNA reads accumulated at 4.5 to 4.8 and 2.6 to 3.3 million in healthy Arapahoe and Mace, respectively (Table 2; Fig. 2). However, the profile of endogenous sRNAs changed as 21 nt became the most predominant in all virus-infected wheat samples, except in temperature-sensitive resistant Mace at 18°C (Table 2; Fig. 2). In single infections, 29 to 39% of endogenous sRNAs comprised 21 nt, followed by 16 to 25% of 24 nt and 15 to 19% of 22 nt (Table 2). In single infections of Arapahoe with WSMV or TriMV at both temperature treatments and in Mace with WSMV at 27°C, the distribution of sRNAs was significantly different from healthy treatments by chi-square analysis, with the largest increases observed in the 21 nt class (Table 2).

**Table 2:** Effect of WSMV and/or TriMV infection on endogenous small RNA reads in wheat cvs. Arapahoe and Mace at 27°C and 18°C*.

Treatment	sRNA	Arapahoe 27°C		Arapahoe 18°C		Mace 27°C		Mace 18°C	
		# of reads	% reads	# of reads	% reads	# of reads	% reads	# of reads	% reads
Healthy	Total	12167243	100	10973426	100	7757611	100	8731550	100
	20	806975	6.6	776564	7.1	794803	10.2	630902	7.2
	21	2715383	22.3	2447074	22.3	1684079	21.8	2059511	23.6
	22	1797953	14.8	1459317	13.3	1162904	15.0	1272687	14.6
	23	1090929	9.0	1036878	9.4	782875	10.1	810199	9.3
	24	4825020	39.7	4459842	40.6	2570900	33.1	3288525	37.7
	25	930984	7.7	793751	7.2	762050	9.8	669726	7.7
WSMV	Total	12894757	100	11060674	100	9234501	100	9985527	100
	20	997758	7.7	683413	6.2	646750	7.0	635663	6.4
	21	4649705	36.1	3813383	34.5	3456090	37.4	2517751	25.2
	22	2368534	18.4	1644453	14.9	1783895	19.3	1331646	13.3
	23	1776698	13.8	1521863	13.8	1293473	14.0	889407	8.9
	24	2271958	17.6	2770447	25.0	1496174	16.2	3869125	38.7
	25	830104	6.4	627114	5.7	558119	6.0	741935	7.4
	p^c^		<0.01		<0.01		<0.01		0.99
TriMV	Total	9281160	100	10459645	100	6344367	100	9684209	100
	20	728801	7.9	640890	6.1	781218	12.3	673938	7.0
	21	3145453	33.9	4101658	39.2	1850161	29.2	2235625	23.1
	22	1732788	18.7	1795401	17.2	1117095	17.6	1509396	15.6
	23	812277	8.8	879397	8.4	592768	9.3	849639	8.8
	24	2214082	23.9	2655923	25.4	1271097	20.0	3688601	38.1
	25	647758	7.0	386376	3.7	732027	11.5	727007	7.5
	p^c^		0.02		<0.01		0.11		0.99
Double	Total	8720262	100	8278569	100	6786552	100	11549340	100
Infection^a^	20	670847	7.7	456764	5.5	445032	6.6	836728	7.2
	21	3207993	36.8	3274999	39.6	2769281	40.8	2772558	24.0
	22	1884811	21.6	1632092	19.7	1422131	21.0	1809250	15.7
	23	1249928	14.3	1168622	14.1	914986	13.5	1117483	9.7
	24	1281383	14.7	1406793	17.0	906697	13.4	4048319	35.1
	25	425299	4.9	339297	4.1	328424	4.8	965001	8.4
	p^c^		<0.01		<0.01		<0.01		0.99

*Small RNA reads were normalized against Arapahoe 18°C (see Table S2).

a Double infection with WSMV (*Wheat streak mosaic virus*) and TriMV (*Triticum mosaic virus*).

b The small RNA pool was analyzed independently for WSMV and TriMV-derived vsiRNAs, and the small RNAs that did not align with the plus- and minus-sense genomic strands of WSMV and TriMV were considered as endogenous sRNAs of wheat.

c Probability (p) of observing a numerically larger Χ^2^ using healthy sRNA distribution as “expected” and treatment sRNA distribution as “observed”.

Similarly, the shift in the profile of endogenous sRNAs was significantly different from healthy in double infections of Arapahoe at both temperature treatments and in Mace at 27°C, with 21 nt sRNAs as the most abundant (37 to 41%), followed by 22 nt (20 to 22%) and 24 nt (13 to 17%) sRNAs (Table 2). Co-infection caused an increase in the accumulation of 21 and 22 nt sRNAs and a decrease in the accumulation of 24 nt sRNAs. In double infections, 24 nt endogenous sRNAs accumulated at 13 to 17% compared with 33 to 41% and 16 to 25% in healthy and single infections, respectively (Table 2; Fig. 2), suggesting that co-infection with WSMV and TriMV caused a drastic reduction in the accumulation of 24 nt endogenous sRNAs in wheat cultivars. Co-infection of Arapahoe at both temperatures and Mace at 27°C caused a statistically significant shift in sRNA accumulation compared to healthy wheat cultivars (Table 2).

In contrast, the profile of endogenous sRNAs in singly and doubly infected Mace at 18°C did not differ significantly from healthy (Table 2; Fig. 2), most likely due to Mace’s resistance to WSMV and TriMV at 18°C. Accordingly, the lack of Mace’s infection at 18°C did not alter the profile of endogenous sRNAs, further supporting that virus infection caused a shift in the profile of endogenous small RNAs.

### Profile of vsiRNAs in WSMV and/or TriMV-infected wheat cultivars

The most prevalent vsiRNAs from WSMV- and/or TriMV-infected Arapahoe at both temperatures and Mace at 27°C were 21 nt (4.5 to 10.2 million reads; 58 to 73%), followed by 22 nt (1.6 to 5.1 million reads; 23 to 39%) (Table 3; Fig. 2). The 20, 23 and 24 nt vsiRNAs accumulated at much reduced levels with only 0.3 to 2.8% (Table 3; Fig. 2). Further examination of vsiRNAs revealed differential accumulation of 21 and 22 nt vsiRNAs in singly and doubly infected Arapahoe (Table 3). In singly infected Arapahoe at 18°C, 21 nt vsiRNAs of WSMV and TriMV accumulated at elevated levels of 72 to 73% compared with 59 to 62% at 27°C. In contrast, in doubly infected plants at 18°C and 27°C, 21 nt vsiRNAs accumulated at approximately similar ratios of 58 to 61% (Table 3). In single infections, 22 nt vsiRNAs accumulated at 23 to 25% at 18°C compared with 34 to 39% at 27°C (Table 3), suggesting that biogenesis of 22 nt vsiRNAs decreased at 18°C in Arapahoe. In contrast, in doubly infected Arapahoe at 18°C and 27°C, 22 nt vsiRNAs accumulated similarly at 34 to 35% (Table 3).

**Table 3:** Percent 20 to 25 nt vsiRNA reads in WSMV and/or TriMV-infected wheat cv. Arapahoe and Mace at 27°C and 18°C*.

Treatment	vsiRNA	Arapahoe: 27°C		Arapahoe: 18°C		Mace: 27°C		Mace: 18°C	
		# of reads	% reads	# of reads	% reads	# of reads	% reads	# of reads	% reads
SI-WSMV	Total	11719186	100	10534114	100	16237784	100	20297	100
	20	208249	1.8	156441	1.5	350719	2.2	1888	9.3
	21	7255942	61.9	7554313	71.7	10165908	62.6	12424	61.2
	22	3948516	33.7	2449786	23.3	5089002	31.3	4968	24.5
	23	163285	1.4	159487	1.5	319513	2.0	500	2.5
	24	126674	1.1	185207	1.8	269499	1.7	350	1.7
	25	16518	0.1	28877	0.3	43140	0.3	164	0.8
DI-WSMV	Total	9862244	100	11723825	100	14555658	100	136839	100
	20	202040	2.0	172514	1.5	303305	2.1	4576	3.3
	21	5887881	59.7	6814228	58.1	8707981	59.8	90624	66.2
	22	3464465	35.1	4072520	34.7	4869706	33.5	37193	27.2
	23	174472	1.8	301952	2.6	354709	2.4	1953	1.4
	24	119529	1.2	324803	2.8	275690	1.9	1894	1.4
	25	13855	0.1	37807	0.3	44265	0.3	596	0.4
SI-TriMV	Total	11114562	100	11107955	100	6348520	100	47806	100
	20	145428	1.3	110105	1.0	150148	2.4	1469	3.1
	21	6521625	58.7	8147094	73.3	4546650	71.6	36088	75.5
	22	4337288	39.0	2739606	24.7	1602136	25.2	9690	20.3
	23	60240	0.5	53246	0.5	25028	0.4	283	0.6
	24	44957	0.4	52122	0.5	21136	0.3	180	0.4
	25	5022	0.0	5781	0.1	3421	0.1	94	0.2
DI-TriMV	Total	7552714	100	8899746	100	9371582	100	76682	100
	20	151851	2.0	120746	1.4	178595	1.9	2312	3.0
	21	4635332	61.4	5345483	60.1	5572734	59.5	53255	69.5
	22	2567021	34.0	3058606	34.4	3167472	33.8	19380	25.3
	23	114389	1.5	172246	1.9	239785	2.6	835	1.1
	24	76433	1.0	183182	2.1	185591	2.0	697	0.9
	25	7686	0.1	19482	0.2	27404	0.3	200	0.3

*^:^The number of vsiRNA reads were normalized against Arapahoe 18°C (see Table S2).

SI: single infection; DI: double infection with WSMV (*Wheat streak mosaic virus*) and TriMV (*Triticum mosaic virus*).

In singly and doubly infected Mace at 18°C, vsiRNAs accumulated to lower levels than in Arapahoe and Mace at 27°C (Fig. 2; Table 3). In singly infected Mace at 18°C, for 21 and 22 nt vsiRNAs, 12,424 and 4968 reads were detected in WSMV-infected, and 36,088 and 9690 reads were detected in TriMV-infected samples, respectively (Table 3). However, in double infections, 21 and 22 nt vsiRNAs accumulated at higher levels at 90,624 and 37,193 of WSMV-derived and 53,255 and 19,380 of TriMV-derived reads, respectively (Table 3). These numbers correspond to a 7-fold increment for WSMV reads and to a 1.5- to 2-fold increment for TriMV reads in doubly infected samples. Reduced levels of vsiRNAs accumulation in Mace at 18°C are consistent with its tolerance to WSMV and TriMV. Hence, reduced levels of viral RNAs were available as a substrate for DCLs to process, which led to basal levels of vsiRNAs.

### Effect of the synergistic interaction between WSMV and TriMV on the biogenesis of vsiRNAs compared with endogenous sRNAs

Accumulation of vsiRNAs and endogenous sRNAs was compared within a sample, but not between the samples because high-throughput sequencing of small RNAs may not reflect accurate quantitative differences between the samples due to variation in the quality and quantity of starting small RNA preparations. Hence, the pool of small RNA reads of deep sequencing most accurately represents differences within a sample but not between the samples.

The effect of the synergistic interaction between WSMV and TriMV on vsiRNA biogenesis was examined by comparing vsiRNAs from singly and doubly infected wheat with those of endogenous sRNAs in Arapahoe and Mace. Accumulation of 20 to 25 nt endogenous sRNAs and vsiRNAs in singly and doubly infected Arapahoe and Mace is presented in Table 4. In singly infected Arapahoe at 18°C and 27°C, WSMV- and TriMV-derived vsiRNAs accumulated to 91 to 95% and 106 to 120% of corresponding endogenous sRNAs, respectively. In contrast, double infections in Arapahoe caused increased accumulation of WSMV-derived vsiRNAs but not those of TriMV when compared with endogenous sRNAs (Table 4). In Mace at 27°C, WSMV- and TriMV-derived vsiRNAs accumulated at 176% and 100%, respectively, of endogenous sRNAs in single infections compared with 215% and 138% in double infections (Table 4). These data suggest that double infections in Arapahoe at 18°C and 27°C caused a significant increase in the accumulation of WSMV-derived vsiRNAs, but little or no increase in TriMV-derived vsiRNAs compared with endogenous sRNAs. In contrast, double infections in Mace at 27°C caused a substantial increase in accumulation of both WSMV- and TriMV-derived vsiRNAs compared with endogenous sRNAs. However, in singly and doubly infected Mace at 18°C, vsiRNAs accumulated at much reduced levels compared with Arapahoe (at 18°C and 27°C) and Mace (at 27°C) (Table 4). A slightly increased level of vsiRNAs accumulation in doubly infected Mace at 18°C compared with those of single infections could be due to marginally increased virus replication in co-infected plants [8].

**Table 4:** WSMV and/or TriMV infection affected biogenesis of vsiRNAs in wheat cv. Arapahoe and Mace at 27°C and 18°C.

Treatment^a^	20 to 25 nt small RNA reads		
	sRNAs^b^	vsiRNAs^c^	% vsiRNAs of sRNAs^d^
Ar-27C-SI-WSMV	12894757	11719186	90.9
Ar-27C-DI-WSMV	8720261	9862243	113.1
Ar-27C-SI-TriMV	9281160	11114562	119.8
Ar-27C-DI-TriMV	8720261	7552714	86.6
Ar-18C-SI-WSMV	11060674	10534114	95.2
Ar-18C-DI-WSMV	8278568	11723824	141.6
Ar-18C-SI-TriMV	10459645	11107955	106.2
Ar-18C-DI-TriMV	8278568	8899745	107.5
Mc-27C-SI-WSMV	9234501	16237783	175.8
Mc-27C-DI-WSMV	6786551	14555657	214.5
Mc-27C-SI-TriMV	6344367	6348519	100.1
Mc-27C-DI-TriMV	6786551	9371582	138.1
Mc-18C-SI-WSMV	9985526	20297	0.2
Mc-18C-DI-WSMV	11549340	136839	1.2
Mc-18C-SI-TriMV	9684208	47806	0.5
Mc-18C-DI-TriMV	11549340	76682	0.7

*Small RNA reads normalized using Arapahoe at 18°C as a standard (see Table S2).

a Ar: Wheat cv. Arapahoe; Mc: Wheat cv. Mace; SI: single infection with either WSMV or TriMV; DI: double infection with WSMV and TriMV.

b small RNAs that are not aligned with virus genomes are considered as endogenous small RNAs.

c virus specific siRNAs to WSMV and TriMV.

d Percent vsiRNAs accumulation compared to endogenous RNAs.

### The polarity of vsiRNAs

**Figure 3:**
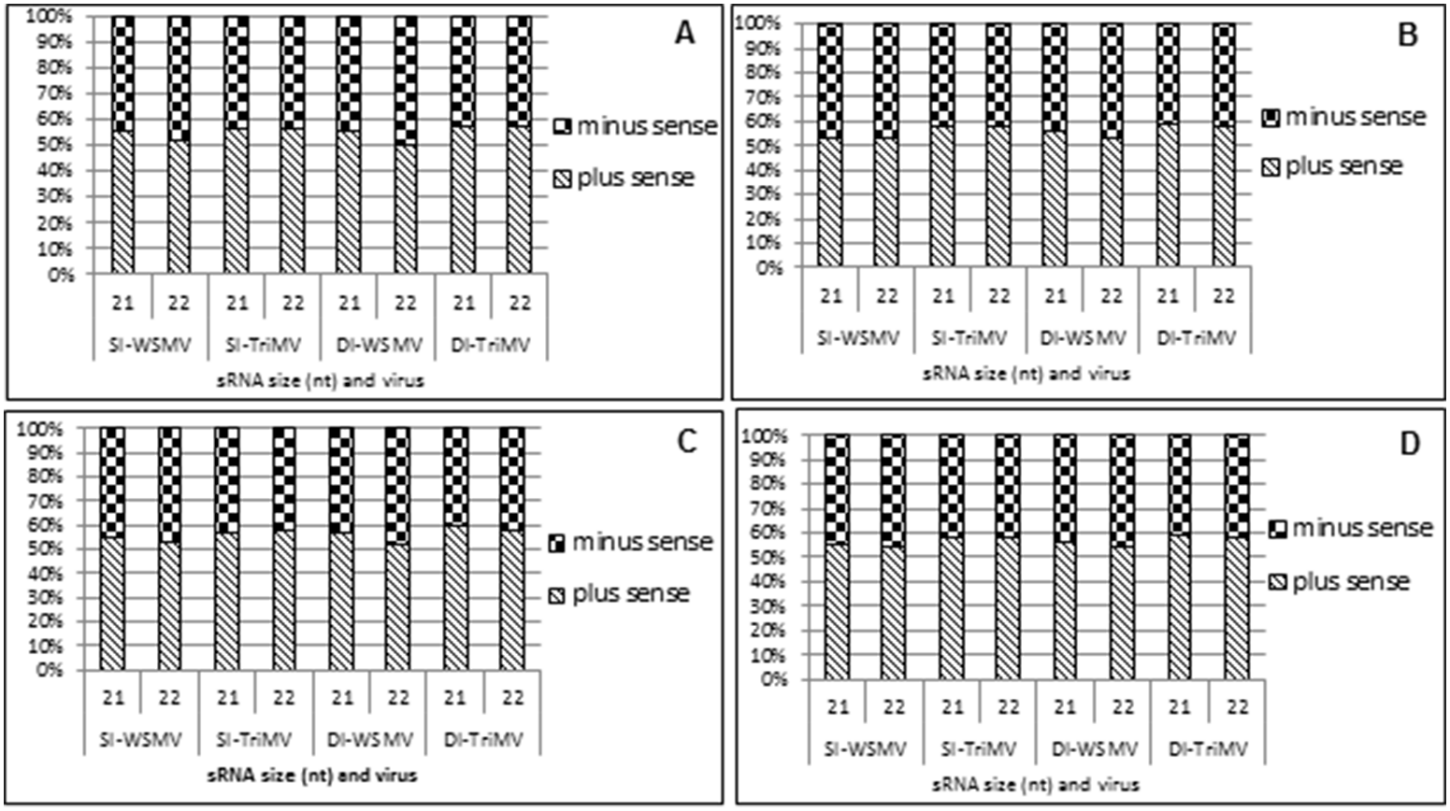
Percent polarity distribution of 21 and 22 nt virus-derived small interfering RNAs (vsiRNAs) in WSMV-, TriMV-, and WSMV plus TriMV-infected wheat cvs. Arapahoe and Mace at 18°C and 27°C. SI: single infection; DI: double infection; A: Arapahoe at 27°C; B: Arapahoe at 18°C; C: Mace at 27°C; D: Mace at 18°C.

The polarity of the most abundant 21 and 22 nt redundant vsiRNAs was analyzed and presented in Figure 3. The plus- and minus-sense polarity of vsiRNAs of all the samples accumulated at 52 to 59% and 41 to 48%, respectively, except for an equimolar accumulation of plus- and minus-sense 22 nt vsiRNAs of WSMV in doubly infected Arapahoe at 27°C (Fig. 3). More plus-sense vsiRNAs accumulated in TriMV-infected samples (56 to 59%) compared to WSMV (50 to 56%) (Fig. 3), suggesting that slightly more vsiRNAs originated from the genomic RNA of TriMV compared to that of WSMV.

Analysis of strand polarity of nonredundant vsiRNAs revealed a different picture from those of redundant vsiRNAs (Table 5). Nonredundant vsiRNAs of WSMV origin accumulated at an equimolar ratio of plus- and minus-sense polarity in single and double infections in Arapahoe at 18°C and 27°C and Mace at 27°C (Table 5). However, nonredundant vsiRNAs of TriMV origin accumulated with a moderate excess of plus-sense polarity (54 to 55%) compared with those of minus-sense polarity (46 to 45%). In Mace at 18°C, fewer WSMV- and TriMV-derived nonredundant vsiRNAs accumulated in singly and doubly infected plants, but vsiRNAs of plus-sense polarity accumulated at 53 to 56% compared with 44 to 47% of minus-sense polarity (Table 5).

**Table 5:** Polarity of nonredundant vsiRNAs from WSMV and/or TriMV-infected wheat.

Treatment^a^	Non-redundant reads	Plus polarity (% reads)	Minus polarity (% reads)
Ar-27°C-SI-WSMV	58572	29269 (50%)	29303 (50%)
Ar-27°C-DI-WSMV	55591	27980 (50%)	27611 (50%)
Ar-18°C-SI-WSMV	61294	30792 (50%)	30502 (50%)
Ar-18°C-DI-WSMV	63370	31782 (50%)	31588 (50%)
Ar-27°C-SI-TriMV	51556	28005 (54%)	23551 (46%)
Ar-27°C-DI-TriMV	51728	28000 (54%)	23728 (46%)
Ar-18°C-SI-TriMV	53085	28751 (54%)	24334 (46%)
Ar-18°C-DI-TriMV	60121	32262 (54%)	27859 (46%)
Mc-27°C-SI-WSMV	65206	32846 (50%)	32360 (50%)
Mc-27°C-DI-WSMV	65298	32862 (50%)	32436 (50%)
Mc-18°C-SI-WSMV	8566	4681 (55%)	3885 (45%)
Mc-18°C-DI-WSMV	17984	9543 (53%)	8441 (47%)
Mc-27°C-SI-TriMV	51582	28152 (55%)	23340 (45%)
Mc-27°C-DI-TriMV	62839	33824 (54%)	29015 (46%)
Mc-18°C-SI-TriMV	10917	6099 (56%)	4818 (44%)
Mc-18°C-DI-TriMV	13404	7535 (56%)	5869 (44%)

a Ar: Arapahoe, Mc: Mace; SI: single-infection, DI: double infection.

### The 5′-terminal nucleotide of 21 and 22 nt vsiRNAs

**Figure 4:**
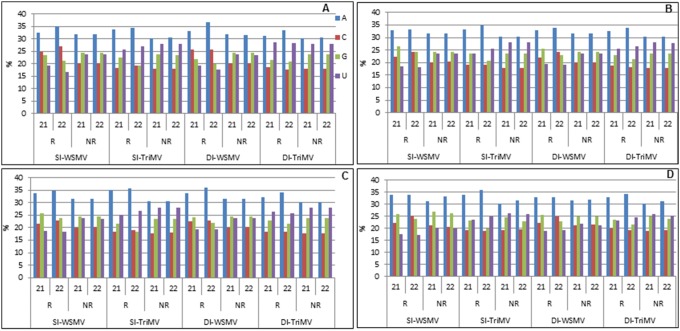
Histograms showing the 5′-terminal nucleotide of 21- and 22 nt redundant (R) and nonredundant (NR) vsiRNAs in WSMV-, TriMV-, and WSMV plus TriMV-infected wheat cvs. Arapahoe and Mace at 18°C and 27°C. SI: single infection; DI: double infection. A: Arapahoe at 27°C; B: Arapahoe at 18°C; C: Mace at 27°C; D: Mace at 18°C.

The most abundant 21 and 22 nt redundant and nonredundant vsiRNAs of WSMV and TriMV were grouped by their 5′-terminal nucleotide (A, C, G, or U) as presented in Figure 4. Redundant vsiRNAs from WSMV- and/or TriMV-infected wheat plants exhibited a clear predominance of A at the 5′ end in 30 to 37% of vsiRNAs (Fig. 4). The second most predominant 5′-terminal nucleotide in the vsiRNAs of TriMV in single and double infections was U in 24 to 28% of the population while nucleotide C was the least predominant 5′-terminal nucleotide in 18 to 20% of vsiRNAs (Fig. 4). In WSMV-specific vsiRNAs, nucleotide C or G was found as the second most predominant 5′- terminal nucleotide in 24 to 27% of vsiRNAs in singly and doubly infected plants (Fig. 4). These data suggest that a modest number of vsiRNAs contain a 5′-terminal nucleotide as an A, and nucleotide C, G, or U was present as a 5′-terminal nucleotide in the remainder of vsiRNAs. However, there was no strong bias toward a particular nucleotide for the 5′-terminal nucleotide.

We also analyzed nonredundant vsiRNAs for the 5′-terminal nucleotide from all the samples. In WSMV- and/or TriMV-infected samples, the predominant 5′-terminal nucleotide of nonredundant 21 and 22 nt vsiRNAs was A (30 to 33%), followed by G (23 to 27%), U (20 to 28%), and C (18 to 22%) (Fig. 4). These data suggest that nucleotide A was moderately predominant and nucleotide C was the least predominant 5′-terminal nucleotide in nonredundant vsiRNAs (Fig. 4).

### Distribution of vsiRNAs in the genomes of WSMV and TriMV

Because a large number of WSMV- and TriMV-derived redundant vsiRNAs accumulated in infected wheat, the distribution of 21 to 24 nt vsiRNAs along the corresponding genomic RNAs of WSMV or TriMV was examined to reveal any hot- and cold-spot regions for vsiRNAs. A region of 5 nt or more without any 21 to 24 nt vsiRNA reads was considered to be a ‘cold-spot’, and a peak with more than 50,000 reads was considered to be a hot-spot for vsiRNAs.

**Figure 5:**
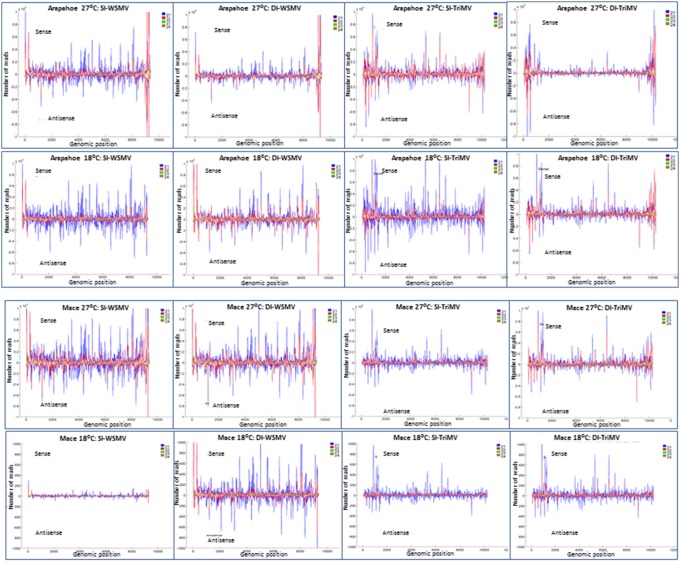
Distribution of 21 to 24 nt redundant vsiRNAs throughout the genomes of WSMV and TriMV in wheat cv. Arapahoe and Mace at 18°C and 27°C. The vsiRNA reads greater than 50,000 were considered as hot-spots. Note that a different scale was used for vsiRNAs in Mace at 18°C.

The genomes of WSMV and TriMV were covered completely with both plus and minus-sense polarity vsiRNAs of 21 to 24 nt in all the samples, except in Mace at 18°C. In Mace at 18°C, fewer vsiRNAs accumulated, especially in single infections with a few cold-spots without vsiRNAs in the genomes of WSMV and TriMV. WSMV-derived vsiRNAs in Mace at 18°C covered most of the genomic RNA except nt 2719, 3503 to 3505, 3843 to 3844, and 5475 to 5478. However, in doubly infected Mace at 18°C, WSMV-derived vsiRNAs covered the entire genomic RNA (Fig. 5). In singly and doubly infected Arapahoe at 18°C and 27°C and Mace at 27°C, WSMV vsiRNAs covered the entire RNA genome with a substantially increased number of vsiRNAs in certain genomic hot-spot regions (see below).

Even though more TriMV-specific vsiRNAs accumulated in singly infected Mace at 18°C compared with those of WSMV (Table 3), six cold-spot regions of a 5 to 28 nt stretch were found in the TriMV genome. The cold-spot regions found in the TriMV genome in singly infected Mace at 18°C corresponded to nt 1 to 28, 1398 to 1418, 3141 to 3155, 7357 to 7362, 7686 to 7690 and 8565 to 8572. The locations of cold-spot regions in the TriMV genome were conserved in singly and doubly infected Mace at 18°C. In TriMV-infected Mace at 18°C, single digit vsiRNA reads were found in nt representing 29 to 87, but fewer vsiRNA reads covered this region in double infections. Though a large number of TriMV-derived vsiRNAs accumulated in singly and doubly infected Arapahoe at 18°C and 27°C and Mace at 27°C, vsiRNAs accumulated only in single-digit numbers in corresponding cold-spot regions found in Mace at 18°C.

Nonredundant vsiRNAs of WSMV and TriMV covered the entire respective genomic RNAs in all the samples, except in singly infected Mace at 18°C (Fig. S1). Similar cold-spot regions were found in the TriMV genome with respect to redundant and nonredundant vsiRNAs.

### Hot-spots for vsiRNAs in the genomes of WSMV and TriMV

In WSMV and/or TriMV-infected Arapahoe at 18°C and 27°C and Mace at 27°C, 6.3 to 16.2 million reads of 20 to 25 nt plus- and minus-sense vsiRNAs accumulated in each virus infected sample (Table 3). Several peaks representing multiple vsiRNA reads were found along the plus and minus-sense genomic RNAs of WSMV and TriMV (Fig. 5). The location of peaks with respect to position in the WSMV or TriMV genome was conserved in singly and doubly infected wheat cultivars at 18°C and 27°C. However, the number of vsiRNA reads varied among different treatments of WSMV or TriMV (Fig. 5).

A clear difference in size and composition of vsiRNA peaks was observed between WSMV-infected Arapahoe 18°C and 27°C samples (Fig. 5). In singly and doubly infected Arapahoe at 27°C, two large vsiRNA peaks were clustered predominantly with 22 nt vsiRNAs toward the 3′ end of the genomic RNA between nt 9100 to 9131 and 9231 to 9291 (Fig. 5). However, much smaller peaks were observed at similar locations with 21 and 22 nt vsiRNAs in Arapahoe at 18°C. Similar hot-spots, as observed in Arapahoe at 27°C, were also found in singly and doubly infected Mace at 27°C (Fig. 5).

Several vsiRNA hot-spots in the TriMV genome were also found in singly and doubly infected Arapahoe at 18°C and 27°C and Mace at 27°C (Fig. 5). The hot-spots were spread throughout the TriMV genome in singly infected Arapahoe at 18°C and 27°C. However, internal hot-spots in the TriMV genome disappeared in doubly infected Arapahoe at 27°C and were reduced to 3 in Arapahoe at 18°C (Fig. 5). In Mace at 27°C, two internal hot-spots were detected in singly infected TriMV, but the number of hot-spots increased toward the termini of genomic RNA in the doubly infected sample (Fig. 5).

In singly and doubly infected Mace at 18°C, fewer WSMV- and TriMV-derived vsiRNAs accumulated due to its temperature sensitive-resistance, and peaks with fewer vsiRNAs were found at the same position as in Mace at 27°C (Fig. 5).

## Discussion

Synergistic interactions among unrelated viruses in plants have been well-documented [2], but the mechanisms behind these interactions have not been deciphered. It has been postulated that the combination of silencing suppressor proteins of two unrelated viruses additively counteract host RNA silencing defense mechanisms, leading to an effective co-invasion of plants by unrelated viruses [2], [5], [6]. In this study, the endogenous sRNA and vsiRNA profiles in singly and doubly infected wheat cultivars were examined in an effort to understand the role of RNA silencing on synergistic interaction between WSMV and TriMV in wheat.

Even though WSMV and TriMV are distantly related type members of two distinct genera in the family *Potyviridae* [47], [48], co-infection of these two viruses interacted synergistically in wheat cultivars Arapahoe and Tomahawk and in Mace at 27°C [8], [52]. We found a large number of WSMV and TriMV-derived vsiRNAs accumulated in singly and doubly infected Arapahoe at both temperatures (18°C and 27°C) and in Mace only at 27°C, indicating that virus infection triggered antiviral RNA silencing responses in these hosts. Additionally, accumulation of abundant vsiRNAs in doubly infected wheat cultivars shows that the presence of two silencing suppressors did not inhibit the biogenesis of virus-derived small RNAs. Our previous work demonstrated that silencing suppressor proteins of WSMV and TriMV repressed host RNA silencing by inhibiting production of vsiRNAs [55], [56]. Taken together, these data suggest that synergism results from events that occur downstream of vsiRNA biogenesis; hence, synergistic interaction and RNA silencing are likely unrelated.

*A. thaliana* and rice encode four and eight DCL proteins, respectively [33], [34]; however, there is no information on the numbers and roles of DCL proteins in wheat. The roles of four *A. thaliana* DCL proteins in the biogenesis of 21 to 24 nt vsiRNAs have been thoroughly characterized [61]: DCL2 and DCL4 produces 22 nt and 21 nt vsiRNAs, respectively, while DCL3 is involved in the biogenesis of 24 nt vsiRNAs in the absence of DCL4 and DCL2 [23], [36]. In healthy Arapahoe and Mace, 24 nt endogenous sRNAs are the most predominant, followed by 21, 22, and 23 nt. However, virus infections caused a dramatic shift in the accumulation of endogenous sRNAs and 21 nt became the most prevalent size class in all hosts, except in Mace at 18°C, which is resistant to infection and accumulates the lowest levels of viral RNA [8], [53]. Virus infections also caused an increased accumulation of 22 nt endogenous sRNAs in singly and doubly infected plants compared to healthy wheat cultivars, except in Mace at 18°C. Our data indicate that WSMV and/or TriMV infections up-regulated production of 21 and 22 nt sRNAs and down-regulated biogenesis of 24 nt endogenous sRNAs. The shift in the profile of endogenous sRNAs is greater in doubly infected than in singly infected plants. These data suggest that in the absence of virus infection, DCL3 is involved in the biogenesis of 24 nt endogenous sRNAs, but it appears that virus infection changes DICER preference to DCL4 and DCL2 for the biogenesis of 21 and 22 nt endogenous sRNAs, respectively. It is conceivable that in healthy plants, DCL1 and DCL3 are active in regulating growth and development, but upon virus infection, plants shift to DCL2 and DCL4 to protect from invading viral pathogens.

Plants have evolved to tolerate biotic and abiotic stress by reprogramming gene expression via the biogenesis of endogenous small RNAs [62]. The up-regulation of endogenous 21 and 22 nt sRNAs and down-regulation of 24 nt sRNAs in virus-infected wheat cultivars suggests that biogenesis of endogenous sRNAs is reprogrammed in virus-infected wheat. This hypothesis is further supported as increased differential accumulation of 21, 22, and 24 nt sRNAs was observed in a doubly infected susceptible wheat cultivar with essentially no change in singly and doubly infected temperature-sensitive resistant wheat cv. Mace at 18°C. It has been reported that stress-induced sRNAs might down-regulate their target genes [63], which may encode negative regulators of stress responses. Conversely, down-regulation of small RNAs in response to stress might cause increased accumulation of their target mRNAs, leading to a positive contribution to stress adaptation. Identification of the target genes for 21, 22, and 24 nt endogenous sRNAs in wheat is beyond the scope of this work. Future research would decipher the target genes of these endogenous sRNAs, enabling better understanding of host-virus interactions. Alternatively, down-regulation of endogenous 24 nt sRNAs could be directly induced by virus infection or could result indirectly from changes in gene expression in response to virus infection.

In RNA silencing, 21 and 22 nt vsiRNAs play a crucial role in combating viral infections [30]. In WSMV- and TriMV-infected plants, 93 to 98% of vsiRNAs are composed of 21 and 22 nt. Double infections in Arapahoe at 18°C and 27°C and Mace at 27°C induced severe symptoms [8] and caused an increased accumulation of vsiRNAs compared to single infections (Fig. 1). In singly and doubly infected Arapahoe, 20 to 25 nt vsiRNAs accumulated at 91 to 120% and 87 to 142% of endogenous sRNAs, respectively, indicating efficient biogenesis of vsiRNAs in doubly infected Arapahoe. In contrast, vsiRNAs in singly and doubly infected Mace at 27°C accumulated at substantially elevated levels of 100 to 176% and 138 to 215%, respectively, of endogenous sRNAs. These data suggest that compared to Arapahoe, the host defense system in Mace at 27°C effectively counteracted invading viruses by processing viral RNAs into vsiRNAs, thus causing a surge in the production of vsiRNAs. These data, together with increased accumulation of vsiRNAs in doubly infected wheat cultivars in Northern blots (Fig. 1) further suggest that synergism and vsiRNA accumulation are most likely not related.

Polarity analysis of redundant vsiRNAs of WSMV and TriMV from singly and doubly infected plants revealed that the plus-sense vsiRNAs were predominant than the minus-sense, suggesting that the majority of vsiRNAs were generated from plus-sense genomic RNAs. In contrast, the plus- and minus-sense nonredundant vsiRNAs exhibited stark differences between WSMV and TriMV infection with symmetrical and asymmetrical accumulation, respectively. The polarity of nonredundant vsiRNAs might give some cues on the vsiRNA biogenesis: WSMV nonredundant vsiRNAs originate from viral dsRNAs and subsequent biogenesis of redundant vsiRNAs might be a combination of primary vsiRNAs from dsRNAs and secondary vsiRNAs by host RDRs. It is possible that TriMV-specific vsiRNAs were generated using the latter method. Several studies indicate that cloned vsiRNAs are predominantly plus-sense polarity in nature compared to those of minus-sense polarity as exemplified in *Turnip mosaic virus* [64], *Potato virus* X [65], and *Citrus tristeza virus* [66]. The polarity of vsiRNAs of *Rice stripe virus* are host dependent [67] and some other viruses produced an approximately equal ratio of plus- and minus-sense polarity [for eg. 68–71].

The 5′-terminal nucleotides of vsiRNAs determine the vsiRNA loading preference into specific AGO complexes: vsiRNAs with a 5′-terminal U nucleotide preferentially load into the AGO1 complex, nucleotide A into AGO2 and AGO4, and C into AGO5 [72]–[75]. The 5′ nucleotide in 32 to 37% of vsiRNAs in singly and doubly infected plants of two wheat cultivars is an A. The 5′-nucleotide of vsiRNAs of WSMV and TriMV approximately followed the nucleotide composition of their genomic RNAs with A(32%)>U(24%)>C(20%)>G(24%) in WSMV and A(30%)>U(28%)>C(18%)>G(24%) in TriMV. Hence, there is no preferential accumulation of vsiRNAs based on 5′ nt. In TriMV and WSMV, vsiRNAs with 5′-nucleotide A and U comprise about 56 to 58%; these vsiRNAs might preferentially load into wheat homologs of AtAGO1, AtAGO2 and AtAGO4.

vsiRNAs were generated from most of the genomic RNAs of WSMV and TriMV in wheat cultivars, suggesting that DCL enzymes target most of genomic RNAs. However, some regions of the WSMV and TriMV genomes were found to be hot-spots for preferential excessive vsiRNA accumulation of more than 50,000 reads. More hot-spot regions in the genomes of TriMV and WSMV were found in singly and doubly infected wheat at 18°C than 27°C, suggesting that DCL enzymes at 18°C preferentially cleave more regions than at 27°C. In addition to hot-spot regions, we also found several cold-spot regions of 5 to 21 nt long in the genomic RNA of TriMV with no vsiRNA reads. In the WSMV genome, though no cold-spot regions were found, some regions had fewer reads than others. The genomic regions with hot- and cold-spots for vsiRNAs might depend on the accessibility of RNA to DCL enzymes, or the visRNAs may preferentially accumulate by associating with AGOs or with the silencing suppressor. The hot-spot regions of genomic RNA might have extensive secondary structures that are favorable for easy access to DCL enzymes. The WSMV and TriMV genomic regions with hot-spot regions were folded into stem-and-loop structures in the mFOLD program (data not shown). However, the *in* *vivo* nature of these secondary structures needs to be verified by probing with nuclease treatment.

## Supporting Information

Figure S1 **Distribution of nonredundant vsiRNAs throughout the genomes of WSMV and TriMV in wheat cv. Arapahoe (a) and Mace (b) at 18°C and 27°C.**
(PPTX)

Table S1 **Total number of small RNA (host and virus-derived) reads and bases obtained by deep sequencing from healthy and WSMV and/or TriMV-infected wheat cultivars.**
(DOCX)

Table S2 **Normalization of small RNAs reads (host and virus) accumulated in healthy and WSMV and/or TriMV-infected wheat cultivars Arapahoe and Mace at 18°C and 27°C against total small RNA reads accumulated in Arapahoe at 18°C.**
(DOCX)
