# Supplementary material for: Dynamics of Small RNA Profiles of Virus and Host Origin in Wheat Cultivars Synergistically Infected by Wheat Streak Mosaic Virus and Triticum Mosaic Virus: Virus Infection Caused a Drastic Shift in the Endogenous Small RNA Profile
Source: PLoS One. 2014 Nov 3;9(11):e111577. doi: 10.1371/journal.pone.0111577 (PMC4218773; doi:10.1371/journal.pone.0111577)
Supplement: Figure S1 — Distribution of nonredundant vsiRNAs throughout the genomes of WSMV and TriMV in wheat cv. Arapahoe (a) and Mace (b) at 18°C and 27°C. (PPTX) [file pone.0111577.s001.pptx]

## Slide 1
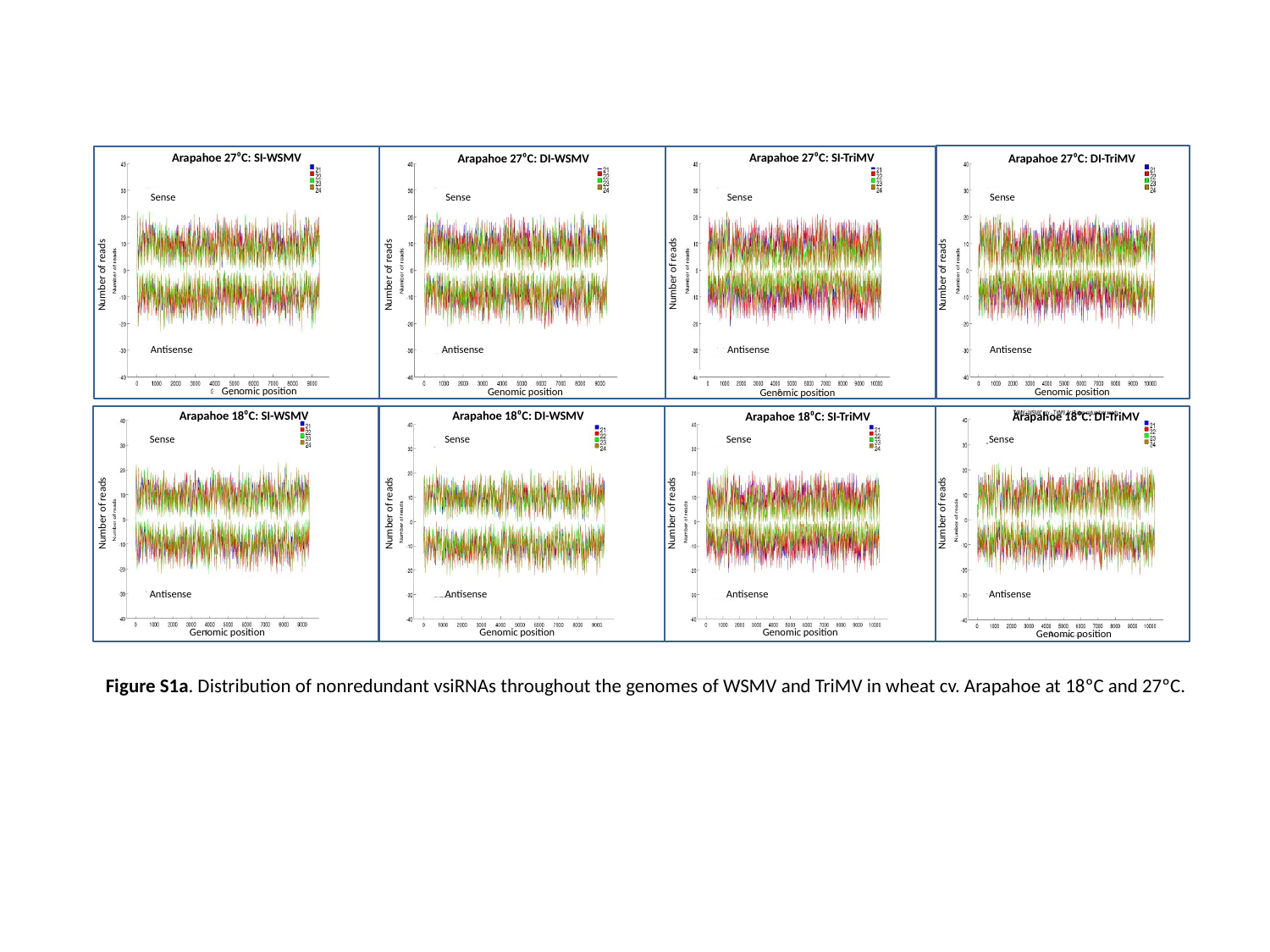

Arapahoe 27⁰C: SI-TriMV
Arapahoe 27⁰C: SI-WSMV
Arapahoe 27⁰C: DI-WSMV
Arapahoe 27⁰C: DI-TriMV
Sense
Sense
Sense
Sense
Number of reads
Number of reads
Number of reads
Number of reads
Antisense
Antisense
Antisense
Antisense
Genomic position
Genomic position
Genomic position
Genomic position
Arapahoe 18⁰C: SI-WSMV
Arapahoe 18⁰C: DI-WSMV
Arapahoe 18⁰C: DI-TriMV
Arapahoe 18⁰C: SI-TriMV
Sense
Sense
Sense
Sense
Number of reads
Number of reads
Number of reads
Number of reads
Antisense
Antisense
Antisense
Antisense
Genomic position
Genomic position
Genomic position
Genomic position
Figure S1a. Distribution of nonredundant vsiRNAs throughout the genomes of WSMV and TriMV in wheat cv. Arapahoe at 18ºC and 27ºC.

## Slide 2
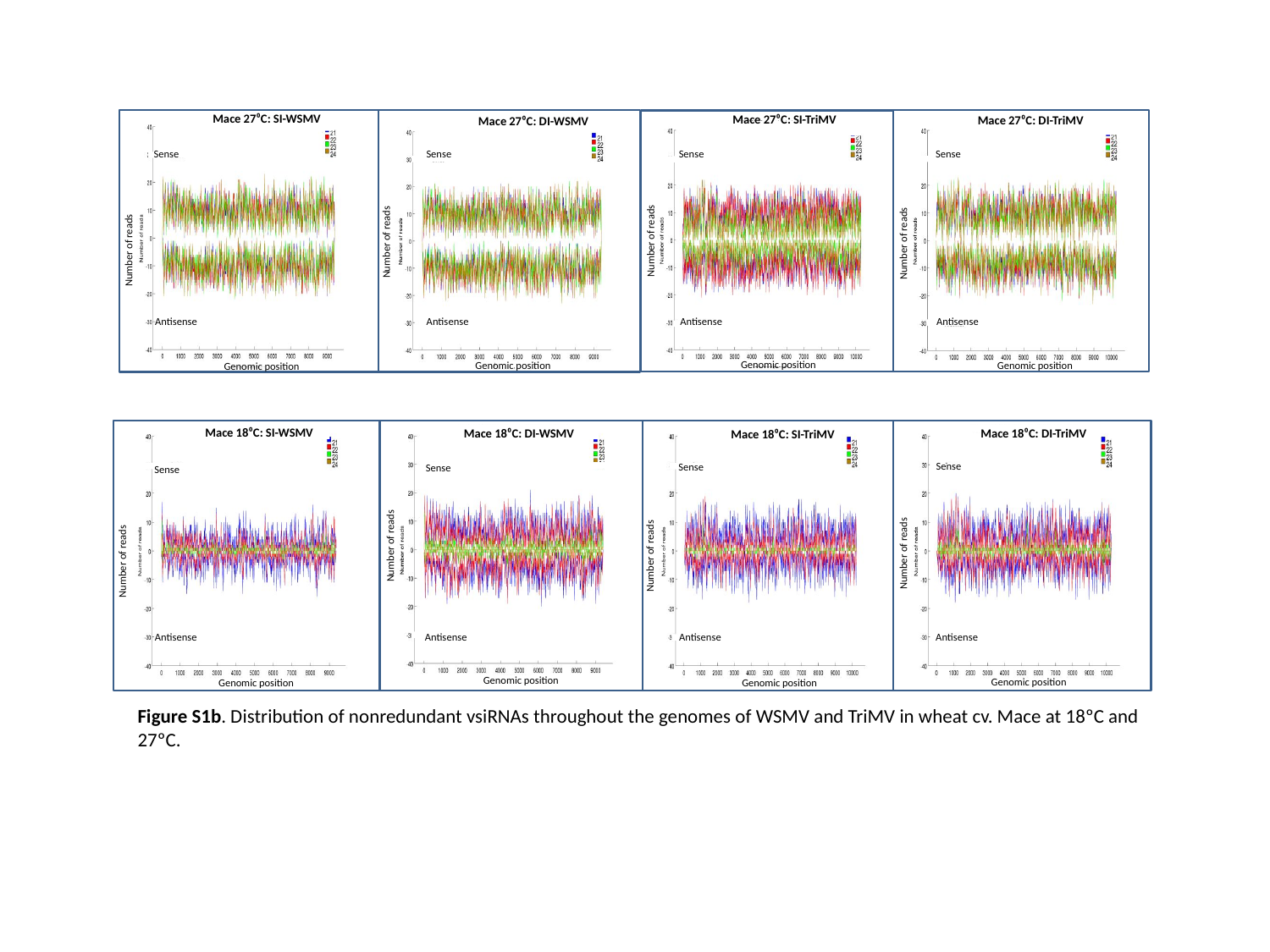

Mace 27⁰C: SI-WSMV
Mace 27⁰C: SI-TriMV
Mace 27⁰C: DI-TriMV
Mace 27⁰C: DI-WSMV
Sense
Sense
Sense
Sense
Number of reads
Number of reads
Number of reads
Number of reads
Antisense
Antisense
Antisense
Antisense
Genomic position
Genomic position
Genomic position
Genomic position
Mace 18⁰C: SI-WSMV
Mace 18⁰C: DI-WSMV
Mace 18⁰C: DI-TriMV
Mace 18⁰C: SI-TriMV
Sense
Sense
Sense
Sense
Number of reads
Number of reads
Number of reads
Number of reads
Antisense
Antisense
Antisense
Antisense
Genomic position
Genomic position
Genomic position
Genomic position
Figure S1b. Distribution of nonredundant vsiRNAs throughout the genomes of WSMV and TriMV in wheat cv. Mace at 18ºC and 27ºC.
