# Supplementary material for: Dynamics of Small RNA Profiles of Virus and Host Origin in Wheat Cultivars Synergistically Infected by Wheat Streak Mosaic Virus and Triticum Mosaic Virus: Virus Infection Caused a Drastic Shift in the Endogenous Small RNA Profile
Source: PLoS One. 2014 Nov 3;9(11):e111577. doi: 10.1371/journal.pone.0111577 (PMC4218773; doi:10.1371/journal.pone.0111577)
Supplement: Table S1 — Total number of small RNA (host and virus-derived) reads and bases obtained by deep sequencing from healthy and WSMV and/or TriMV-infected wheat cultivars. (DOCX) [file pone.0111577.s002.docx]

| Sample | Number of Reads | Total Bases |
| --- | --- | --- |
| Ar 18 | 39,352,733 | 1,028,143,088 |
| Ar 27 | 34,431,333 | 877,348,058 |
| Ma 18 | 38,124,697 | 1,024,696,936 |
| Ma 27 | 37,745,815 | 919,539,552 |
| Ar 18 WSMV | 36,559,768 | 891,060,970 |
| Ar 27 WSMV | 37667851 | 906052244 |
| Ma 18 WSMV | 36579349 | 951769816 |
| Ma 27 WSMV | 37500390 | 854080729 |
| Ar 18 TriMV | 36324551 | 886678591 |
| Ar 27 TriMV | 30663323 | 743704643 |
| Ma 18 TriMV | 36496702 | 955228883 |
| Ma 27 TriMV | 35369287 | 841846587 |
| Ar 18 WSMV+TriMV | 33784820 | 775725345 |
| Ar 27 WSMV + TriMV | 30210869 | 684722624 |
| Ma 18 WSMV + TriMV | 32392404 | 913470351 |
| Ma 27 WSMV + TriMV | 35129928 | 785695509 |
|  |  |  |
| Total | 568,333,820 | 14,039,763,926 |

Table S1. Total number of small RNA (host and virus-derived) reads and bases obtained by deep sequencing from healthy and WSMV and/or TriMV-infected wheat cultivars.

Ar: Wheat cultivar Arapahoe; MA: Wheat cultivars Mace; 18: Plants incubated at 18ºC; 27: plants incubated at 27ºC.
