# Supplementary material for: Dynamics of Small RNA Profiles of Virus and Host Origin in Wheat Cultivars Synergistically Infected by Wheat Streak Mosaic Virus and Triticum Mosaic Virus: Virus Infection Caused a Drastic Shift in the Endogenous Small RNA Profile
Source: PLoS One. 2014 Nov 3;9(11):e111577. doi: 10.1371/journal.pone.0111577 (PMC4218773; doi:10.1371/journal.pone.0111577)
Supplement: Table S2 — Normalization of small RNAs reads (host and virus) accumulated in healthy and WSMV and/or TriMV-infected wheat cultivars Arapahoe and Mace at 18°C and 27°C against total small RNA reads accumulated in Arapahoe at 18°C. (DOCX) [file pone.0111577.s003.docx]

| Description | Total Number of reads | Normalization factor |
| --- | --- | --- |
| Arapahoe 18ºC | 39309484 | 1.000 |
| Arapahoe 27ºC | 34301010 | 1.146 |
| Mace 18ºC | 38095968 | 1.032 |
| Mace 27ºC | 37714903 | 1.042 |
| Ar 18ºC-WSMV | 36523144 | 1.076 |
| Ar 27ºC -WSMV | 37644014 | 1.044 |
| Ma 18ºC- WSMV | 36343728 | 1.082 |
| Ma 27ºC- WSMV | 37475000 | 1.049 |
| Ar 18ºC -TriMV | 36305953 | 1.083 |
| Ar 27ºC- TriMV | 30644540 | 1.283 |
| Ma 18ºC- TriMV | 36462928 | 1.078 |
| Ma 27ºC-TriMV | 35333897 | 1.113 |
| Ar 18ºC -WSMV + TriMV | 33726241 | 1.166 |
| Ar 27ºC -WSMV + TriMV | 29746114 | 1.321 |
| Ma 18ºC -WSMV + TriMV | 32366800 | 1.215 |
| Ma 27ºC- WSMV + TriMV | 35104004 | 1.120 |

Table S2. Normalization of small RNAs reads (host and virus) accumulated in healthy and WSMV and/or TriMV-infected wheat cultivars Arapahoe and Mace at 18ºC and 27ºC against total small RNA reads accumulated in Arapahoe at 18ºC.

Ar: Arapahoe; Ma: Mace
